# P-2155. Evaluation of Intracranial Hemorrhage in patients with Infections related to Left Ventricular Assist Device (LVAD) at our Transplant Center

**DOI:** 10.1093/ofid/ofaf695.2318

**Published:** 2026-01-11

**Authors:** Rafael Garcia-Sturgill, Alexander Tarr, Sarah Kanell, George Bchech, Nitin Bhanot, Rasha Abdulmassih

**Affiliations:** Allegheny Health Network, Pittsburgh, Pennsylvania; Allegheny Health Network, Pittsburgh, Pennsylvania; Allegheny Health Network, Pittsburgh, Pennsylvania; Allegheny Health Network, Pittsburgh, Pennsylvania; Allegheny Health Network, Pittsburgh, Pennsylvania; Allegheny Health Network / Drexel University College of Medicine, Pittsburgh, Pennsylvania

## Abstract

**Background:**

Prior studies have shown that elevated international normalized ratio (INR), blood stream infection (BSI) and driveline infection are significant risk factors for intracranial hemorrhage (ICH) in patients with LVAD. Staphylococcus aureus and Pseudomonas aeruginosa infection in particular have previously linked to increased risk of ICH.

We aimed to evaluate patients with LVADs who sustained ICH and determine the possible role of infection contributing to this phenomenon.

**Methods:**

A retrospective analysis was conducted at our institution on patients with LVADs who developed ICH from 2015 to 2024. We collected patient demographics, INR levels, as well as history of LVAD associated infection, antimicrobial use, and presence of active infection at the time of the ICH.

**Results:**

21 patients were identified. The median age was 66 years old, all of them were male. 80% of patients (17/21) had hypertension, 47% had CKD (10/21). 8/21 patients had concern of active infection at time of the event, 4 of them had BSI. (2 Corynebacterium *s*triatum, 1 Pseudomonas aeruginosa, 1 Klebsiella pneumoniae). 12/21 patients had previous BSI and/or driveline associated infection, 8 of them were on suppressive antibiotics prior to admission. 11/21 patients were on antibiotics at the time of the ICH.

Of the identified pathogens, 66% were gram positive. Of these, 5 were Corynebacterium spp. All patients were on warfarin and 47% were on aspirin. The average INR on the day of the bleed was 2.12. Cerebral angiogram was done on 11/21 patients that did not identify any mycotic aneurysm. Echocardiogram was done on 13/21 patients, and no endocarditis was identified in patients with BSI.

**Conclusion:**

While previously identified pathogens (S. aureus, P. aeruginosa) were present, a notable proportion of patients had Corynebacterium, particularly C. striatum. Although differentiating its role as a pathogen versus skin flora is difficult, our findings suggest a potential association with increased ICH risk in this population.

**Disclosures:**

All Authors: No reported disclosures

